# Relationship between Body Mass Index and Bone Turnover Markers in Girls with Idiopathic Central Precocious Puberty

**DOI:** 10.1155/2023/6615789

**Published:** 2023-04-28

**Authors:** Jing Zhang, Wen-jie Zhou, Yi-duo Zhang, Chuan-jiao Liu, Fan Yu, Yong-mei Jiang

**Affiliations:** ^1^Department of Laboratory Medicine, West China Second University Hospital, Sichuan University, Chengdu, China; ^2^Key Laboratory of Birth Defects and Related Diseases of Women and Children, Ministry of Education, West China Second University Hospital, Sichuan University, Chengdu, China; ^3^Department of Laboratory Medicine, Maternal and Child Health Hospital of Qingbaijiang District in Chengdu, Chengdu, China

## Abstract

**Background:**

This study aimed to determine the effect of body mass index (BMI) on bone turnover markers in girls with idiopathic central precocious puberty (ICPP) according to weight status at diagnosis.

**Methods:**

Two hundred and eleven girls with ICPP were divided according to their weight status at diagnosis into three groups: normal weight, overweight, and obese. The serum levels of total procollagen type 1 N-terminal propeptide (P1NP), N-terminal midfragment of osteocalcin, *β*-C-terminal telopeptide of type 1 collagen, and some biochemical indicators were measured. Associations between variables were evaluated by multiple regression analysis.

**Results:**

Serum P1NP concentrations were significantly different among groups (*p* < 0.001). No other significant differences were noted in N-terminal midfragment of osteocalcin and *β*-C-terminal telopeptide of type 1 collagen. BMI was associated with estradiol (*r* = 0.155, *p* < 0.05) and inversely associated with P1NP (*r* = −0.251, *p* < 0.01), luteinizing hormone peak (*r* = −0.334, *p* < 0.01), follicle-stimulating hormone peak (*r* = −0.215, *p* < 0.01), and luteinizing hormone/follicle-stimulating hormone peak (*r* = −0.284, *p* < 0.01). Multiple regression analysis of factors associated with BMI showed that it was correlated with P1NP, follicle-stimulating hormone base, and luteinizing hormone peak in the overweight group and the obese group.

**Conclusions:**

Our findings showed that BMI was associated with P1NP, revealing the reduction of bone formation in overweight and obese girls with ICPP. During the diagnosis and treatment of girls with ICPP, attention should be paid to body weight and bone metabolism.

## 1. Introduction

With changes in lifestyle and diet, the incidence of obesity has risen sharply worldwide [1]. According to worldwide data from the Global Health Observatory, 18% of youth aged 5 to 19 years were overweight or obese in 2016 [2, 3]. Evidence is mounting that obesity will decrease the age that puberty starts, thus making obesity a risk factor for precocious puberty [4]. Recently, concern has grown that obesity may negatively affect bone development in children. Evidence exists that links excess fat to bone-related diseases such as osteoporosis and skeletal fractures [5]. Both adipose and bone tissue come from the same progenitor mesenchymal cells, which are endocrine organs capable of producing hormones [5]. The connection between adipose and bone tissue seems to cause confusion, stemming from the fact that obesity is both a protective state and a risk factor for osteoporosis [6]. The most recent evidence indicates that increased body mass index (BMI) translates to higher bone mineral density (BMD); however, the parallel increase in adiposity alters bone-regulating hormones that can diminish bone quality [7].

Idiopathic central precocious puberty (ICPP) is the most common form of precocious puberty in girls, and it often leads to adverse effects on physical and psychological maturity [8]. Precocious puberty is a complex biological process of sexual development in which bones are observed to mature prematurely and rapidly. When abnormal hormones affect bone quality in children with precocious puberty, there may be a subsequent impact on their bone quality in adulthood, especially considering the potentially lower BMD and the risks of osteoporosis and fractures [9].

During the growth process, bone turnover markers (BTMs) may act as sensitive markers for dynamic observation of the bone turnover process and early detection of abnormal bone mass changes. Among such markers, procollagen type I N-terminal propeptide (P1NP) and N-terminal midfragment of osteocalcin (N-MID) reflect bone formation, while *β*-C-terminal telopeptide of type 1 collagen (*β*-CTX) reflects bone resorption [10]. Bone mineral mass changes are influenced by various hormones, which directly or indirectly regulate bone physiology [11]. Their levels may also provide information about bone metabolism [12, 13]. A major determinant of osteoporosis is the magnitude of peak bone mass achieved in early adulthood. Peak bone mass, together with the bone size, geometry, and microstructure achieved in children and youth, constitutes total bone strength and resistance to fractures in the later stages of life [14–16]. Therefore, it is very important to focus on the changes in bone metabolism in children and adolescents diagnosed with ICPP.

However, there are few reports on how BMI affects BTMs in girls with ICPP. To understand whether BMI affects BTMs, we investigated the relationship between BMI and BTMs in 211 girls diagnosed with ICPP in our center. We hope that this analysis will help in the diagnosis and treatment decisions for girls with ICPP and improve the bone metabolism of girls with different levels of obesity.

## 2. Methods

### 2.1. Participants

Two hundred and eleven girls diagnosed with ICPP aged 5–10 years participated in this study. They were recruited from the outpatient clinic unit of West China Second University Hospital from March 1, 2020, to October 31, 2021. All participants were confirmed to show signs of secondary sexual development, such as breast enlargement, before the age of 8 years, and none of the participants had received treatment. The individuals were divided into groups according to BMI as follows: normal weight, 5–10 years (*n* = 89); overweight, 6–10 years (*n* = 70); and obesity, 5–9 years (*n* = 52). The evaluation criteria for normal weight, overweight, and obesity were a BMI value between the 5th percentile and <85th percentile of normal girls of the same age, a BMI value between the 85th percentile and the <95th percentile of normal girls of the same age, and a BMI value for ≥95th percentile of normal girls of the same age, respectively [17]. Height and weight were measured by a nurse. The height measurement did not include shoes, and the weight measurement did not include clothing and shoes. Tanner stage ratings 1 through 5 were assigned by clinicians. Tanner stage was used only for descriptive purposes and was not included in the models to adjust for growth/puberty. Instead, we used age to adjust for growth.

### 2.2. Inclusion Criteria

All of the girls included in the study met the diagnostic criteria of ICPP [18]. The diagnosis of ICPP requires the following conditions to be met simultaneously: (1) development of secondary sex characteristics before the age of 8 years in girls and 9 years in boys, with the same sequence of sexual development as that in children with normal sexual development; (2) a higher annual growth rate compared with children of the same age and sex; (3) higher bone age relative to the chronological age by at least 1 year; (4) increased uterus and ovaries, and more than one ovarian follicle with a diameter of ≥4 mm on pelvic ultrasound in girls; and (5) hypothalamic-pituitary-gonadal axis activation.

To determine whether hypothalamic-pituitary-gonadal axis function is activated, a gonadotropin-releasing hormone (GnRH) excitation test is required to achieve a luteinizing hormone (LH) peak ≥5.0 U/L and an LH/follicle-stimulating hormone (FSH) peak ratio >0.6. Participants with secondary obesity, such as hypercortisolism or primary thyroid obesity caused by low function, who had previously received hormone drugs or were receiving drugs for weight loss, diabetes mellitus, and other chronic liver and kidney diseases were excluded. The study protocol was approved by the Clinical Trial Ethics Committee of West China Second University Hospital, Sichuan University (No. 2020040). Informed consent was obtained from the parents of all of the girls before enrollment. All clinical investigations were conducted in accordance with the principles outlined in the Declaration of Helsinki.

### 2.3. Collection of Blood Samples and Measurement of Biomarkers

The GnRH stimulation test was performed before diagnosis in all children, and fasting venous blood samples were collected before the GnRH test and 30 minutes, 60 minutes, 90 minutes, and 120 minutes after the start of the GnRH test. Blood samples were collected at 9:00 am after a fasting period of 8 hours for the detection of BTMs and biochemical indicators. Aliquots of 4 mL of whole blood were placed in a dry tube with no anticoagulant for serum separation. Serum aliquots were immediately stored at −80°C until further analysis. BTMs, including P1NP, N-MID, and *β*-CTX, were measured with a Cobas E411 electrochemical luminescence immunity analyzer (Roche, Mannheim, Germany). Serum insulin-like growth factor 1 (IGF1) and insulin-like growth factor binding protein-3 (IGFBP-3) levels were determined using an IMMULITE 2000 chemiluminescence analyzer (Siemens AG FWB, Berlin, Germany). An ADVIA Centaur XP system (Siemens AG FWB) was used to measure the serum levels of free triiodothyronine, free thyroxine, triiodothyronine, thyroxine, thyroid-stimulating hormone (TSH), LH, FSH, and estradiol (E2). Serum alkaline phosphatase, fasting blood glucose, homocysteine, total cholesterol, triglyceride, high-density lipoprotein cholesterol, and low-density lipoprotein cholesterol were measured using an ADVIA 2400 system (Siemens AG FWB). Serum vitamin D was evaluated using an Abbott i2000SR immunoluminescence detector (Abbott Laboratories, Chicago, IL, USA). The analytical variability of the BTMs, including intra-assay and interassay coefficients of variation, was all less than 8.0%. The total coefficients of variation for E2, LH, and FSH were 1.9%–7.0%, 2.7%–3.8%, and 2.2%–3.9%, respectively.

### 2.4. Statistical Analysis

Data were analyzed using SPSS software version 23.0 (IBM, Armonk, NY, USA). Descriptive statistics are presented as median and quartiles for non-normally distributed characteristics. Comparison between groups was conducted using the Kruskal–Wallis *H* test. Additionally, Kolmogorov–Smirnov tests were used to compare any two groups. Chi-square tests were used for comparison of count data. Spearman correlation analysis was performed to examine the correlations among data. Associations between variables were evaluated using a multiple regression analysis. A value of *p* < 0.05 was considered significant for all the statistical analyses.

## 3. Results

### 3.1. Baseline Characteristics

For participants, the following clinical data were collected from the medical records, namely, chronological age, height, weight, and bone age at the time of diagnosis. A total of 211 girls with ICPP were included, of which girls with normal weight, overweight, and obesity accounted for 42.2%, 33.2%, and 24.6% of the sample, respectively. No differences between groups were found in chronological age. The normal weight group was shorter compared with the overweight group (*p* < 0.05) and the obese group (*p* < 0.05). There were significant differences in weight between the normal weight group and the overweight and obese groups (*p* < 0.05). The bone age in the obese group was more advanced than that in the normal weight group (*p* < 0.05) and the overweight group (*p* < 0.05) (Table 1). The number of participants stratified by Tanner staging is shown in Table 1. All participants showed Tanner breast scores ≥2 before the age of 8 years. General characteristics of the participants are shown in Table 1, and general characteristics of ungrouped ICPP girls are shown in Supplementary Table 1.

### 3.2. Bone Turnover Markers Analysis

Results from the analyses of BTMs are presented in Table 2. The levels of P1NP measured in blood were significantly different among groups (*p* < 0.001). Levels of P1NP in the normal weight group were significantly higher than those in the overweight and obese groups (*p* < 0.05). P1NP levels in the obese group were significantly lower than those in the overweight group (*p* < 0.05). However, no other significant differences were noted in N-MID and *β*-CTX. BTM levels for the participants are shown in Supplementary Table 1.

### 3.3. Blood Biochemical Characteristics

There were significant differences in IGF1, IGFBP-3, homocysteine, free triiodothyronine, free thyroxine, TSH, LH base, LH peak, FSH peak, and LH/FSH peak among the three groups (*p* < 0.05). We did not find specific alterations of alkaline phosphatase, fasting blood glucose, thyroxine, high-density lipoprotein cholesterol, and FSH base concentrations in any group. The serum levels of free thyroxine, TSH, vitamin D, LH peak, FSH peak, and LH/FSH peak were significantly different in the normal weight group compared with the overweight group. Serum IGF1, IGFBP-3, free triiodothyronine, triglyceride, low-density lipoprotein cholesterol, E2, LH peak, FSH peak, and LH/FSH peak levels in the normal weight group were also different from those in the obese group. In addition, we found that IGFBP-3, homocysteine, triiodothyronine, TSH, total cholesterol, triglyceride, E2, LH base, LH peak, and LH/FSH peak concentration were significantly different between the overweight group and obese group.  Table 3 shows the differences between any two groups of results. The blood biochemical characteristics of participants are shown in Supplementary Table 1.

### 3.4. The Associations between BMI and BTMs as well as Hormones

In all participants, BMI was inversely associated with P1NP (*r* = −0.251, *p* < 0.01; Table 4). In addition, BMI was associated with most hormones. BMI was also associated with E2 (*r* = 0.155, *p* < 0.05) and FSH base (*r* = 0.184, *p* < 0.01), as well as inversely associated with LH peak (*r* = −0.334, *p* < 0.01), FSH peak (*r* = −0.215, *p* < 0.01), and LH/FSH peak (*r* = −0.284, *p* < 0.01). P1NP was inversely related to E2 (*r* = −0.152, *p* < 0.05), and N-MID was positively correlated to the FSH peak (*r* = 0.163, *p* < 0.05). LH base was positively correlated to P1NP, and FSH base was positively correlated to N-MID.

### 3.5. Regression Analysis of Factors Associated with BMI

**Figure 1:**
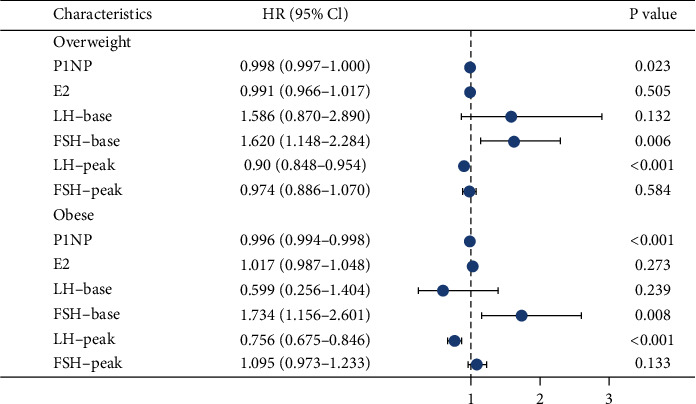
Multiple regression analysis of factors associated with body mass index.

We performed a multiple regression analysis of the variables that are affected by BMI (Figure 1). BMI was correlated with P1NP, FSH base, and LH peak in the overweight and obese groups.

## 4. Discussion

The International Osteoporosis Foundation and the International Federation of Clinical Chemistry and Laboratory Medicine strongly recommend *β*-CTX and P1NP as BTMs for clinical research. *β*-CTX in the circulation is greatly affected by circadian rhythm and food intake. Serum P1NP is not significantly affected by food but is affected by small circadian rhythms, drugs that stimulate bone growth, sex hormones, and glucocorticoid treatments. Therefore, we measured BTMs in the morning on an empty stomach.

Evidence is inconsistent regarding the association between BMI and BTMs. Some studies have reported increases in BTMs in children and young adults with obesity [19, 20], but others have reported reductions in BTMs with obesity [21]. Further studies have pointed out that the BTMs of children with obesity are significantly lower than those of children with normal weight [22]. Our results show that the levels of P1NP measured in blood were significantly different among groups, but no significant differences were noted in N-MID. The results of the multiple regression analysis showed that BMI was correlated with P1NP. Our findings suggest that children with ICPP in overweight and obese groups have reduced bone formation. Two potential reasons exist for this finding. First, studies show that both adipocytes and osteoblasts derive from multipotent mesenchymal stem cells. Obesity diverts mesenchymal stem cell differentiation toward the adipocyte line and damages osteoblasts. This imbalance leads to a decrease in bone formation [23]. Second, the diet of children with obesity is usually high in fat, which may reduce intestinal calcium absorption, leading to lower calcium availability for bone formation [24].

Studies have shown that the adipose tissue of children with obesity expresses many adipokines, which can downregulate osteoblasts, osteocytes, and muscle cells and upregulate osteoclasts [25]. Our findings show that regardless of which two groups were compared, no significant differences were noted in *β*-CTX. This result seems to be inconsistent with the upregulation of osteoclasts. BMD is affected by many factors during the teenage years, and genetic factors account for 60–80% of BMD [26]. In addition, the hormonal milieu and individual and environmental modifiable factors, such as body weight, obesity, diet, and physical activity, also play an important role in BMD later in life [27, 28]. Therefore, the reason for this inconsistency cannot be simply attributed to a specific reason, and further research is needed.

Our study shows that the LH peak of children with overweight and obesity was lower than that of children with normal weight, and the difference was statistically significant. Spearman correlation analysis shows that the LH peak and FSH peak are negatively correlated with BMI. The multiple regression analysis also showed that BMI was significantly correlated with the FSH base and LH peak. This finding is consistent with that of related research [29], but different from the findings of Giabicani et al. [30]. Currently, the mechanisms underlying decrease in LH and FSH levels with increased BMI are unclear. Moreover, we observed an association between LH base with P1NP and FSH base, and FSH peak with N-MID. Previous reports on elderly individuals, adolescents, and collegiate athletes failed to observe any association between BTMs and FSH or LH [31, 32]. One report on healthy Chinese females found that both FSH and LH were negatively related to bone mass [33]. The discrepancy between our results and previous reports may be explained by differences in ethnicity, age, and the impact of the disease.

The present study has some limitations related to its cross-sectional design and small sample size. BTMs are affected by many factors, such as the region, movement, and nutritional characteristics. In future studies, we will conduct more experiments to verify whether weight has an effect on the reduction of bone formation in girls with ICPP. Moreover, there is still a lack of reference values for BTMs in healthy children in China. Therefore, our follow-up research will also establish a reference value for children's bone metabolism, and further analyze the factors that affect children's bone metabolism. The exclusion of boys owing to the absence of available blood samples may have introduced bias. After the use of GnRH analogs to treat ICPP, there may be a disproportionate increase in BMI [34]. Therefore, it is also important to evaluate the correlation between BMI and bone metabolism in patients after GnRH treatment in future studies. The present study also did not involve specific segmentation of the Tanner staging of the girls because of the limited sample size. Therefore, further well-designed studies are warranted to confirm the present findings.

## 5. Conclusion

The main finding of the present study is that BMI is associated with P1NP, which reveals the reduction of bone formation in overweight and obese girls with ICPP. Obesity may be related to bone metabolism in girls with ICPP. Overall, when diagnosing precocious puberty, the status of patients with obesity should be considered as an influencing factor of puberty hormone concentration, and the degree of obesity should be evaluated when assessing precocious puberty. Being overweight and obese may adversely influence bone formation in girls with ICPP. In the treatment process for ICPP in girls, more attention should be paid to weight management.

## Figures and Tables

**Table 1:** General characteristics of the participants.

Characteristic	Normal weight (*n* = 89)	Overweight (*n* = 70)	Obese (*n* = 52)	*p* value
CA (years)	8.0 (7.0–9.0)	8.0 (8.0–9.0)	8.0 (7.0–9.0)	0.514
Height (cm)	130.80 (127.95–136.25)^a^	135.30 (130.10–139.60)	135.05 (132.00–140.50)^c^	0.001
Weight (kg)	26.50 (24.75–30.00)^a^	32.00 (30.00–35.00)^b^	35.40 (33.00–40.00)^c^	<0.001
BA (years)	10.00 (9.00–10.70)	10.40 (9.70–10.90)^b^	10.65 (10.20–11.40)^c^	<0.001
Tanner 2 (%)	74.16 (66/89)	60.00 (42/70)	50.00 (26/52)	0.038
Tanner 3 (%)	22.47 (20/89)	37.14 (26/70)	42.31 (22/52)	
Tanner 4 (%)	3.37 (3/89)	2.86 (2/70)	7.69 (4/52)	

^a^Significantly different from the overweight group. ^b^Significantly different from the obese group. ^c^Significantly different from the normal weight group. CA, chronological age; BA, bone age.

**Table 2:** Levels of bone turnover markers in different groups.

BTMs	Normal weight (*n* = 89)	Overweight (*n* = 70)	Obese (*n* = 52)	*p* value
P1NP (ng/ml)	994.60 (867.30–1123.50)^a^	950.40 (791.25–1081.00)^b^	712.20 (619.40–1063.00)^c^	<0.001
N-MID (pg/ml)	76.47 (62.98–90.19)	71.79 (60.42–93.61)	74.18 (62.20–84.20)	0.486
*β*-CTX (ng/ml)	1560.00 (1170.00–1806.00)	1540.00 (1234.00–1760.00)	1425.00 (957.20–1850.00)	0.713

^a^Significantly different from the overweight group. ^b^Significantly different from the obese group. ^c^Significantly different from the normal weight group. BTMs, bone turnover markers; P1NP, total procollagen type 1 N-terminal propeptide; N-MID, N-terminal midfragment of osteocalcin; *β*-CTX, *β*-C-terminal telopeptide of type 1 collagen.

**Table 3:** Blood biochemical characteristics of participants.

Items	Normal weight (*n* = 89)	Overweight (*n* = 70)	Obese (*n* = 52)	*p* value
ALP (U/L)	295.00 (259.00–352.00)	294.50 (254.00–365.00)	319.50 (246.00–366.00)	0.840
FBG (mmol/L)	4.61 (4.42–4.82)	4.63 (4.39–4.83)	4.70 (4.54–4.81)	0.417
IGF1 (ng/mL)	281.00 (207.50–342.50)	311.00 (235.00–389.00)	340.50 (273.00–381.00)^c^	0.002
IGFBP-3 (*μ*g/mL)	5.69 (5.19–6.35)	5.90 (5.37–6.57)^b^	6.58 (5.94–7.04)^c^	<0.001
HCY (*μ*mol/L)	9.00 (7.85–10.15)	9.60 (8.20–10.70)^b^	8.35 (6.90–9.70)	0.011
FT3 (pmol/L)	6.07 (5.80–6.42)	6.18 (5.86–6.49)	6.38 (5.99–6.68)^c^	0.037
FT4 (pmol/L)	15.34 (14.47–17.16)^a^	14.95 (13.74–15.90)	15.16 (13.69–16.73)	0.046
T3 (nmol/L)	2.21 (2.04–2.45)	2.25 (1.95–2.52)^b^	2.30 (2.11–2.61)	0.148
T4 (nmol/L)	107.90 (93.50–123.85)	104.10 (88.70–115.10)	103.20 (92.30–111.70)	0.115
TSH (mIU/L)	2.33 (1.74–3.34)^a^	1.87 (1.25–2.58)^b^	2.69 (1.94–3.47)	0.001
TC (mmol/L)	3.78 (3.50–4.12)	3.73 (3.28–4.48)^b^	3.83 (3.64–4.47)	0.169
TG (mmol/L)	0.80 (0.68–0.96)	0.86 (0.68–1.01)^b^	0.82 (0.70–1.50)^c^	0.073
HDL-C (mmol/L)	1.50 (1.35–1.81)	1.46 (1.30–1.69)	1.47 (1.25–1.68)	0.415
LDL-C (mmol/L)	2.11 (1.82–2.41)	2.11 (1.71–2.73)	2.24 (1.95–2.67)^c^	0.088
VitD (ng/mL)	20.40 (17.45–23.45)^a^	18.40 (15.00–23.60)	21.75 (15.60–24.60)	0.274
E2 (pg/ml)	22.80 (15.00–32.90)	21.00 (16.20–34.30)^b^	26.40 (21.40–33.15)^c^	0.073
LH-base (IU/L)	0.80 (0.50–1.20)	0.90 (0.60–1.50)^b^	0.50 (0.30–1.10)	0.005
FSH-base (IU/L)	2.40 (1.40–3.40)	2.90 (1.50–3.70)	2.35 (2.00–3.30)	0.205
LH-peak (IU/L)	17.10 (12.35–23.10)^a^	13.60 (9.00–18.70)^b^	10.20 (8.20–14.20)^c^	<0.001
FSH-peak (IU/L)	15.40 (12.25–18.20)^a^	13.00 (10.60–16.30)	12.45 (11.00–15.60)^c^	0.014
LH/FSH peak	1.10 (0.89–1.52)^a^	0.90 (0.82–1.29)^b^	0.79 (0.65–0.95)^c^	<0.001

^a^Significantly different from the overweight group. ^b^Significantly different from the obese group. ^c^Significantly different from the normal weight group. ALP, alkaline phosphatase; FBG, fasting blood glucose; IGF1, insulin-like growth factor 1; IGFBP-3, insulin-like growth factor binding protein-3; HCY, homocysteine; FT3, free triiodothyronine; FT4, free thyroxine; T3, triiodothyronine; T4, thyroxine; TSH, thyroid-stimulating hormone; TC, total cholesterol; TG, triglyceride; HDL-C, high-density lipoprotein cholesterol; LDL-C, low-density lipoprotein cholesterol; VitD, vitamin D; E2, estradiol; LH-base, luteinizing hormone base; FSH-base, follicular stimulating hormone base; LH-peak, luteinizing hormone peak; FSH-peak, follicular stimulating hormone peak; LH/FSH peak, luteinizing hormone/follicular stimulating hormone peak.

**Table 4:** Associations of body mass index with other parameters.

Items	BMI	P1NP	N-MID	*β*-CTX
BMI	—			
P1NP	−0.251^*∗∗*^	—		
N-MID	−0.066	0.423^*∗∗*^	—	
*β*-CTX	−0.059	0.306^*∗∗*^	0.403^*∗∗*^	—
E2	0.155^*∗*^	−0.152^*∗*^	0.006	−0.066
LH-base	−0.075	0.180^*∗∗*^	0.094	0.015
FSH-base	0.184^*∗∗*^	−0.006	0.207^*∗∗*^	0.107
LH-peak	−0.334^*∗∗*^	0.078	0.098	0.122
FSH-peak	−0.215^*∗∗*^	0.063	0.163^*∗*^	0.119
LH/FSH peak	−0.284^*∗∗*^	0.046	0.007	0.065

^*∗*^ *p* < 0.05, ^*∗∗*^*p* < 0.01, and *p* < 0.05 were considered statistically significant. BMI, body mass index; P1NP, total procollagen type 1 N-terminal propeptide; N-MID, N-terminal midfragment of osteocalcin; *β*-CTX, *β*-C-terminal telopeptide of type 1 collagen; E2, estradiol; LH-base, luteinizing hormone base; FSH-base, follicular stimulating hormone base; LH-peak, luteinizing hormone peak; FSH-peak, follicular stimulating hormone peak; LH/FSH peak, luteinizing hormone/follicular stimulating hormone peak.

## Data Availability

The data used to support the findings of this study are included in the article. Due to the sensitivity of the data and the restrictions from the informed consent, the data will not be stored at a public repository but are available from the corresponding author upon reasonable request.

## Additional Points

*What Is Known*? Idiopathic central precocious puberty (ICPP) results from premature activation of the hypothalamic-pituitary-gonadal axis before the age of 8 years in girls and 9 years in boys. Obesity is a risk factor for precocious puberty and body mass index (BMI) is associated with bone mineral density and bone quality. There are few reports on how BMI affects bone turnover markers (BTMs) in girls with ICPP. *What Is New*? The levels of total procollagen type 1 N-terminal propeptide (P1NP) were significantly different among normal weight group, overweight group, and obese group. BMI was associated with P1NP, revealing the reduction of bone formation in overweight and obese girls with ICPP. During the diagnosis and treatment of girls with ICPP, attention should be paid to body weight and bone metabolism.

## Supplementary Materials

Supplementary Materials Supplementary Table 1: General characteristics, bone turnover markers, and blood biochemical characteristics in ICPP girls.
