# Supplementary material for: Relationship between Body Mass Index and Bone Turnover Markers in Girls with Idiopathic Central Precocious Puberty
Source: Int J Clin Pract. 2023 Apr 28;2023:6615789. doi: 10.1155/2023/6615789 (PMC10162872; doi:10.1155/2023/6615789)
Supplement: Supplementary Materials — Supplementary Table 1: General characteristics, bone turnover markers, and blood biochemical characteristics in ICPP girls. [file 6615789.f1.docx]

**Supplementary Table**

Supplementary Table 1 General characteristics, bone turnover markers and blood biochemical characteristics in ICPP girls

| Items | ICPP group (n=211) |
| --- | --- |
| CA (years) | 8.0 (7.0–9.0) |
| Height (cm) | 133.50 (129.40–138.00) |
| Weight (kg) | 31.00 (26.50–34.60) |
| BA (years) | 10.40 (9.60–10.90) |
| Tanner 2(%) | 63.51 (134/211) |
| Tanner 3(%) | 32.23 (68/211) |
| Tanner 4(%) | 4.27 (9/211) |
| P1NP (ng/ml) | 948.80 (731.60–1064.00) |
| N-MID (pg/ml) | 74.61 (62.20–88.83) |
| β-CTX (ng/ml) | 1540.00 (1190.00–1802.00) |
| ALP (U/L) | 301.00 (254.00–363.00) |
| FBG (mmol/L) | 4.64 (4.42–4.82) |
| IGF1 (ng/mL) | 306.00 (231.00–373.00) |
| IGFBP-3 (μg/mL) | 5.93 (5.37–6.72) |
| HCY (μmol/L) | 9.10 (7.90–10.10) |
| FT3 (pmol/L) | 6.22 (5.87–6.49) |
| FT4 (pmol/L) | 15.05 (14.02–16.63) |
| T3 (nmol/L) | 2.25 (2.06–2.49) |
| T4 (nmol/L) | 105.90 (92.30–117.40) |
| TSH (mIU/L) | 2.26 (1.57–3.04) |
| TC (mmol/L) | 3.80 (3.41–4.42) |
| TG (mmol/L) | 0.81 (0.69–1.07) |
| HDL-C (mmol/L) | 1.48 (1.30–1.70) |
| LDL-C (mmol/L) | 2.15 (1.88–2.63) |
| VitD (ng/mL) | 20.10 (16.80–23.60) |
| E2 (pg/ml) | 23.40 (17.30–32.90) |
| LH-base (IU/L) | 0.80 (0.50–1.30) |
| FSH-base (IU/L) | 2.60 (1.60–3.50) |
| LH-peak (IU/L) | 14.20 (9.50–18.80) |
| FSH-peak (IU/L) | 14.00 (11.20–17.20) |
| LH/FSH peak | 0.94 (0.75–1.30) |

Abbreviations: CA, chronological age; BA, bone age; P1NP, total procollagen type 1 N-terminal propeptide; N-MID, N-terminal midfragment of osteocalcin; β-CTX, β-C-terminal telopeptide of type 1 collagen; ALP, alkaline phosphatase; FBG, fasting blood glucose; IGF1, insulin-like growth factor 1; IGFBP-3, insulin-like growth factor binding protein-3; HCY, homocysteine; FT3, free triiodothyronine; FT4, free thyroxine; T3, triiodothyronine; T4, thyroxine; TSH, thyroid-stimulating hormone; TC, total cholesterol; TG, triglyceride; HDL-C, high density liptein cholesterol; LDL-C, low-density lipoprotein cholesterol; VitD, vitamin D; E2, estradiol; LH-base, luteinizing hormone base; FSH-base, follicular stimulating hormone base; LH-peak, luteinizing hormone peak; FSH-peak, follicular stimulating hormone peak; LH/FSH peak, luteinizing hormone/follicular stimulating hormone peak.
